# Anti-glioblastoma Activity of Kaempferol via Programmed Cell Death Induction: Involvement of Autophagy and Pyroptosis

**DOI:** 10.3389/fbioe.2020.614419

**Published:** 2020-12-10

**Authors:** Suqin Chen, Jing Ma, Liu Yang, Muzhou Teng, Zheng-Quan Lai, Xiaoyu Chen, Jingjin He

**Affiliations:** ^1^The Eighth Affiliated Hospital, Sun Yat-sen University, Shenzhen, China; ^2^School of Basic Medical Sciences, Southern Medical University, Guangzhou, China; ^3^Department of Pharmacy, Shenzhen University General Hospital, Shenzhen University, Shenzhen, China; ^4^Department of Neurology, The Eighth Affiliated Hospital, Sun Yat-sen University, Shenzhen, China

**Keywords:** kaempferol, glioblastoma, ROS, autophagy, pyroptosis

## Abstract

Glioblastoma is one of the most common and lethal intracranial malignant, and is still lack of ideal treatments. Kaempferol is a major nutrient found in various edible plants, which has exhibited the potential for the treatment of glioblastoma. However, the specific anti-glioma mechanism of kaempferol is yet to be studied. Herein, we aim to explore the mechanisms underlying the anti-glioma activity of kaempferol. Our results demonstrated that kaempferol suppresses glioma cell proliferation *in vitro* and inhibits tumor growth *in vivo*. Moreover, kaempferol raises ROS and decreases mitochondrial membrane potential in glioma cells. The high levels of ROS induce autophagy then ultimately trigger the pyroptosis of glioma cells. Interestingly, when we used 3-MA to inhibit autophagy, we found that the cleaved form of GSDME was also decreased, suggesting that kaempferol induces pyroptosis through regulating autophagy in glioma cells. In conclusion, this study revealed kaempferol possesses good anti-glioma activity by inducing ROS, and subsequently leads to autophagy and pyroptosis, highlighting its clinical potentials as a natural nutrient against glioblastoma.

## Introduction

Glioblastoma (GBM) is one of the most aggressive type of cancers in central nervous system with poor prognosis. Patients diagnosed with GBM have the median survival time range from 5 to 15 months while the 5-year survival rates range from 0 to 5% (Wen and Kesari, 2008; Tsitlakidis et al., 2010; Ostrom et al., 2017). Despite tremendous effort has been devoted to develop novel cancer therapies, the treatment of GBM remained relatively unchanged for decades and consists of surgical resection followed by adjuvant chemoradiotherapy. Currently, Temozolomide (TMZ), a first-line drug in the treatment of GBM, is the most effective regimen that increases the median overall survival of GBM patients from 12 to 14.6 months, and increases the 2-year survival rate from 10.4 to 26.5%(Stupp et al., 2005). However, tumors will inevitably recur in most of cases and seem to be resistant to even higher dose of chemotherapy regimens (Stupp et al., 2009; Gilbert et al., 2013). Besides, traditional chemotherapy drugs usually cause certain side effects. Therefore, it is urgently needed to develop natural anti-tumor drugs with unique curative and low toxicity side effects.

**Figure 1:**
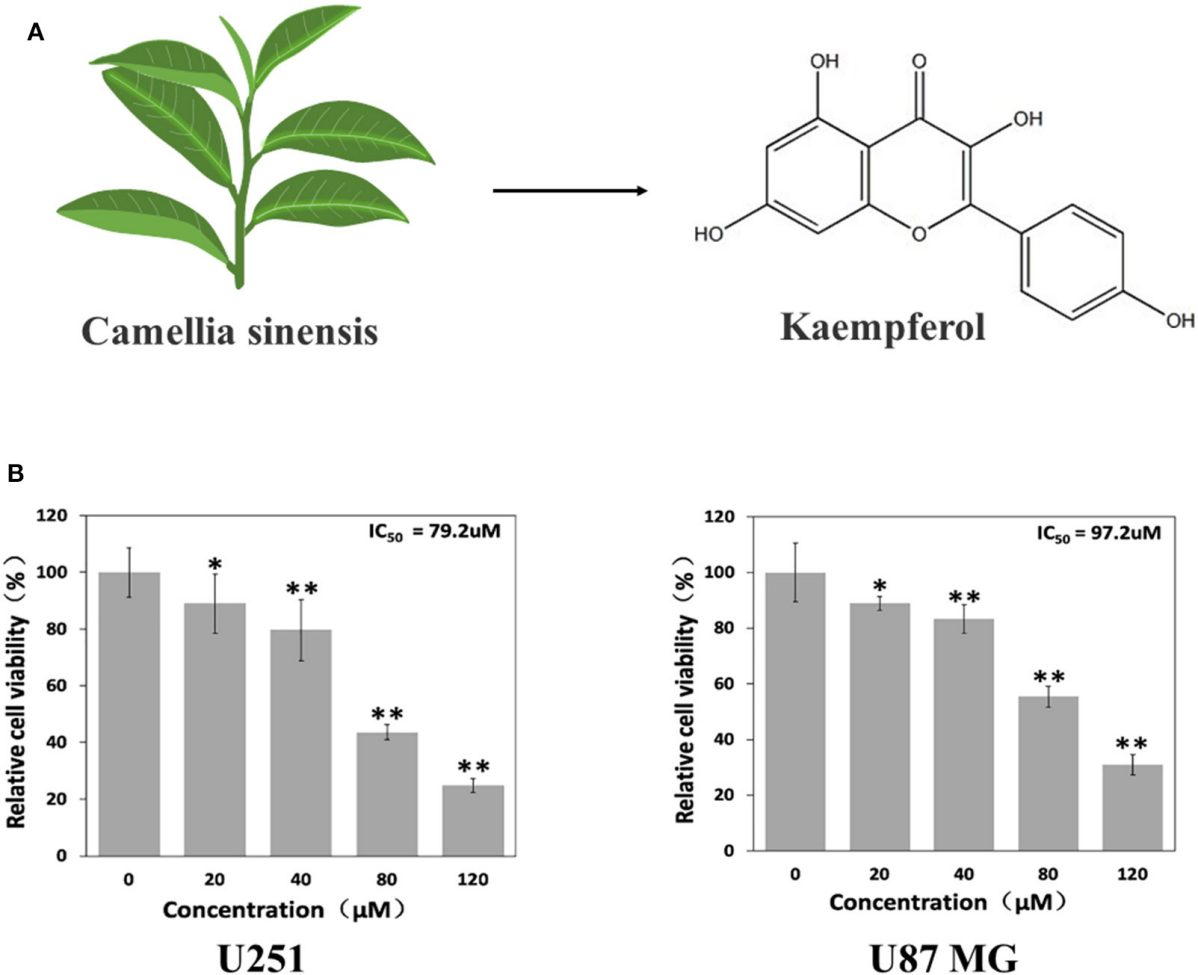
Kaempferol suppressed glioma cells. **(A)** Structure of kaempferol. **(B)** U251 and U87 MG cells were treated with different concentrations of kaempferol for 24 h, and cell viability was assessed by MTT assay, *n* = 6. Data was presented as mean ± SD. *P < 0.05, **P < 0.01 compared with the control.

A large number of natural products derived from plants have shown great promise in treating or preventing cancer in recent years. Flavonoids is a type of natural compounds found abundantly in many herbal medicines which possesses multiple pharmacological activities. Kaempferol is one of the most common aglycone flavonoids in the form of glycoside. It can be found in many edible plants, such as tea (Figure 1A), most vegetables and fruits. Kaempferol has a variety of beneficial biological properties (Harborne and Williams, 2000; Rajendran et al., 2014; Imran and Salehi, 2019), including anti-oxidant, anti-carcinogenic, and anti-inflammatory. According to epidemiologic studies, high intake of kaempferol is associated with decreased risk of different types of cancers (Garcia-Closas et al., 1999; Nöthlings et al., 2007; Cui et al., 2008). Although kaempferol has shown anti-glioma effects *in vitro* (Sharma et al., 2007; Siegelin et al., 2008; Jeong et al., 2009), its *in vivo* anti-glioma activity and the specific mechanism have not been fully elucidated yet.

As we all know, there are several cell death types in cancer treatment, including necrosis, apoptosis, necroptosis, autophagy and pyroptosis. Pyroptosis is an inflammatory form of programmed cell death activated by some inflammasomes. Gasdermin D and Gasdermin E, as the essential mediators of pyroptosis, can be separately cleaved by caspase-1/4/5/11 and caspase-3 into GSDMD-NT and GSDME-NT, which further connects to the cell membranes and triggers oligomerization, forming the pores and leading the leakage of inflammatory substances into intercellular space (Schroder and Tschopp, 2010; Kayagaki et al., 2015; Fang et al., 2020). The activation of gasdermin E in chemotherapy can change the morphology of the dying tumor cells from apoptosis into a pyroptosis-like death (Wang et al., 2017). Autophagy is a process that cells use to recycle intracellular components to sustain metabolism and survival by which cellular material is delivered to lysosomes for degradation. Although autophagy normally promotes cell survival and prevents cancer development (Galluzzi et al., 2015), in advanced cancers, both enhancing and inhibiting autophagy can kill cancer cells. Moreover, inhibiting different stages of autophagy may have the opposite effect in a same cancer (Amaravadi et al., 2016). Autophagy can either promote or inhibit apoptosis under different cellular contexts, the mechanisms underlying these opposing effects are related to the degradation of different pro-apoptotic or anti-apoptotic regulators by autophagy.

It has been reported that increasing intracellular oxidative stress and inducing apoptosis were considered to be one of the anti-glioma mechanisms of kaempferol (Sharma et al., 2007; Jeong et al., 2009). Since ROS could induce a variety of complex cascades, including autophagy and pyroptosis. Therefore, in this study, we aimed to explore the anti-glioma effect of kaempferol *in vitro* and *in vivo*, and further investigate the mechanism of inducing glioma cell death, especially the involvement of autophagy and pyroptosis.

## Materials and Methods

### Reagents

Kaempferol (≥97%, HPLC grade), Thiazolyl Blue Tetrazolium Bromide (MTT), and 3-methyladenine (3-MA) were purchased from Sigma-Aldrich Chemical (St. Louis, MO, USA). 2′, 7′-Dichlorodihydrofluorescein diacetate (H2DCFDA), Hoechst 33342, N-acetyl-L-cysteine (NAC), Lyso-Tracker Green, and MitoTracker Red CMXRos were obtained from Beyotime Biotechnology (Shanghai, China). Annexin V-FITC Apoptosis Detection kit was provided by BD Biosciences (Franklin Lake, New Jersey, USA). Necrosulfonamide (NSA) was obtained from MCE (New Jersey, USA).

### Animals and CDX Model

The anti-glioma effect of kaempferol *in vivo* was conducted on 6-week old male immune-deficient BALB/c Nude Mice (purchased from the laboratory of Southern medical University). Mice were kept in SPF level animal room. All the investigations were approved by the Animal Care and Institutional Ethics Committee of Sun-Yat Sen University and conformed to the US National Institutes of Health (Bethesda, MD, USA) Guide for the Care and Use of Laboratory Animals and the ARRIVE guidelines.

Six BALB/c Nude Mice were randomly divided into two groups (with Two biological repetitions), and U87 MG cells (10^7^/100 μL PBS/mouse) were injected subcutaneously to establish cell line derived xenografts (CDX) models. Mice were checked every other day to monitor the tumor growth and the weight. Then the kaempferol treatment group were received kaempferol (40 mg/kg, dissolved in plant oil) by gavage daily for 3 weeks, while the control group only received the plant oil instead. When the experiment was terminated, mice were euthanized by overdose anesthesia. The tumor tissues and the viscera were removed for further analysis.

### Cell Culture

U87 MG and U251 Glioblastoma cell lines were obtained from the American Type Culture Collection (Rockville, MD, USA) and cultured with DMEM (Gibco BRL, Invitrogen, Carlsbad, CA, USA) containing 10% FBS (Gibco BRL, Invitrogen, Australia, USA) at 37°C with a 5% CO_2_–humidified atmosphere.

### Measurement of Cell Viability

Cell viability was evaluated using MTT assay as previous described (Liu et al., 1997). U87 MG and U251 cells were seeded on 96-well-plates at a density of 5.0 × 10^3^ cells/well. After adhesion, cells were treated with different concentrations (0, 20, 40, 80, and 120 μM) of kaempferol for 24 h. After washing the cells, culture medium containing 0.5 mg/ml of MTT was added to each well. Then the cells were incubated for 4 h at 37°C, the supernatant was removed and the formed formazan crystals in viable cells were solubilized with 100 μL of dimethyl sulfoxide and the absorbance of each well was measured at 570 nm with microplate Reader (Varioskan Lux, Thermo Scientific, USA). Values were expressed as a percentage relative to those obtained in controls. The ratio of viability of control cells in the absence of kaempferol was taken as 100%.

### Measurement of Mitochondrial Membrane Potential

Mitochondrial membrane potential was measured using JC-1 probe. For flow cytometric analysis of JC-1 Staining, U87 MG and U251 cells were cultured in 6-well-plates and treated with different concentrations (0, 40, and 80 μM) of kaempferol for 24 h. Then, the cells were collected and stained with 500 μL 1 × JC-1 staining solution and 500 μL of DMEM for 30 min at room temperature, and the stained cells were tested using a BD LSRFortessa flow cytometer with emission at 590 and 529nm. JC-1 accumulates as J-aggregates (590 nm; red) only in metabolically active mitochondria and depolarization of mitochondrial membranes leads to JC-1 monomer formation (527 nm; green). For fluorescence image assay, U87 MG and U251 cells were cultured in cover glass bottom dishes and treated with 80 μM of kaempferol for 24 h. After stimulation, the cells were stained with JC-1 staining solution (1×) for 30 min and then observed and photographed using a Confocal microscope (LSM 880 with fast Airyscan, Zeiss, Germany).

### Measurement of ROS

Intracellular ROS generation in cells treated with kaempferol was assessed using H2DCFDA probe. After treating with 80 μM of kaempferol for 24 h, Cells cultured in cover glass bottom dishes were incubated with 10 μM H2DCFHDA for 30 min at 37°C and washed twice with PBS. Then cells were observed and photographed using a Confocal microscope (LSM 880 with fast Airyscan, Zeiss, Germany).

### Assays for Mitochondrial Membrane Potential and Autophagy

U87 MG and U251 cells were cultured in cover glass bottom dishes overnight before treating with or without kaempferol (80 μM) for 24 h. Then, the cells were stained with MitoTracker Red CMXRos working solutions (100 nM) and LysoTracker Green working solutions (100 nM) for 1 h. After that, the cells were stained with Hoechst working solutions (1×) or 4′,6′-diamidino-2-phenyl-indole (DAPI) working solutions (1×) for another 20 min. Then, the cells were imaged with a confocal microscope (LSM 880 with fast Airyscan, Zeiss, Germany).

### Western Blotting

Western blotting was used to determine the protein expression level. U87 MG and U251 cells cultured in 6-well-culture plates were treated with different concentrations (0, 40, and 80 μM) of kaempferol for 48 h. The samples of whole-cell protein were prepared with radioimmunoprecipitation assay (RIPA) buffer containing protease/phosphatase inhibitor cocktail (CST), separated by 10–15% sodium dodecyl sulfate polyacrylamide gel electrophoresis, and transferred to nitrocellulose membranes (Millipore). Antibodies including PARP (46D11, CST), LC3 (3868s, CST), γH2AX (9718s, CST), β-actin (4967s, CST), p62/SQSTM1 (18420-1-AP, Proteintech), α-tubulin (5886s, CST), and gasdermin E (GSDME) (ab215191, Abcam) were used in this study. After addition of chemiluminescence reagent (Thermo), the blots were exposed to ChemiDoc Touch imaging System (Singapore) and the images were analyzed using Image J version 1.52 (National Institutes of Health).

### Real-Time-Polymerase Chain Reaction

The mRNA expressions were detected using quantitative polymerase chain reaction (qPCR) assay. Briefly, total RNA was isolated using RNeasy Mini Kit (Qiagen) and reversely transcribed to cDNA via PrimerScript RT reagent Kit with gDNA Eraser (Takara). The primer sequences used in qPCR are as follows, IL-1β: 5′-AGCTACGAATCTCCGACCAC-3′ and 5′-CGTTATCCCATGTGTCGAAGAA-3′; ASC: 5′-TGGATGCTCTGTACGGGAAG-3′ and 5′-CCAGGCTGGTGT-GAAACTGAA-3′. We used TB Green Premix (Takara) to performed qPCR according to the directions.

### Statistical Analysis

Analyses were managed using SPSS version 17.0 (USA). Data in this study were presented as mean ± standard deviation (SD) and analyzed using the *T*-test analysis or Repeated Measures ANOVA. ^*^P < 0.05 was considered to be statistically significant. All experiments were performed in triplicate at a minimum.

## Result

### Kaempferol Suppressed Human GBM Cells *in vitro*

To evaluate whether kaempferol affects viability of GBM cells, MTT assay was done on U87MG and U251 cells. As shown in Figure 1B, kaempferol showed a dose-dependent manner in inhibiting GBM proliferation. The half maximal inhibitory concentration (IC50) of kaempferol against U87MG and U251 cells was 97.2 and 79.2 μM, respectively.

### Kaempferol Suppressed GBM Growth *in vivo*

**Figure 2:**
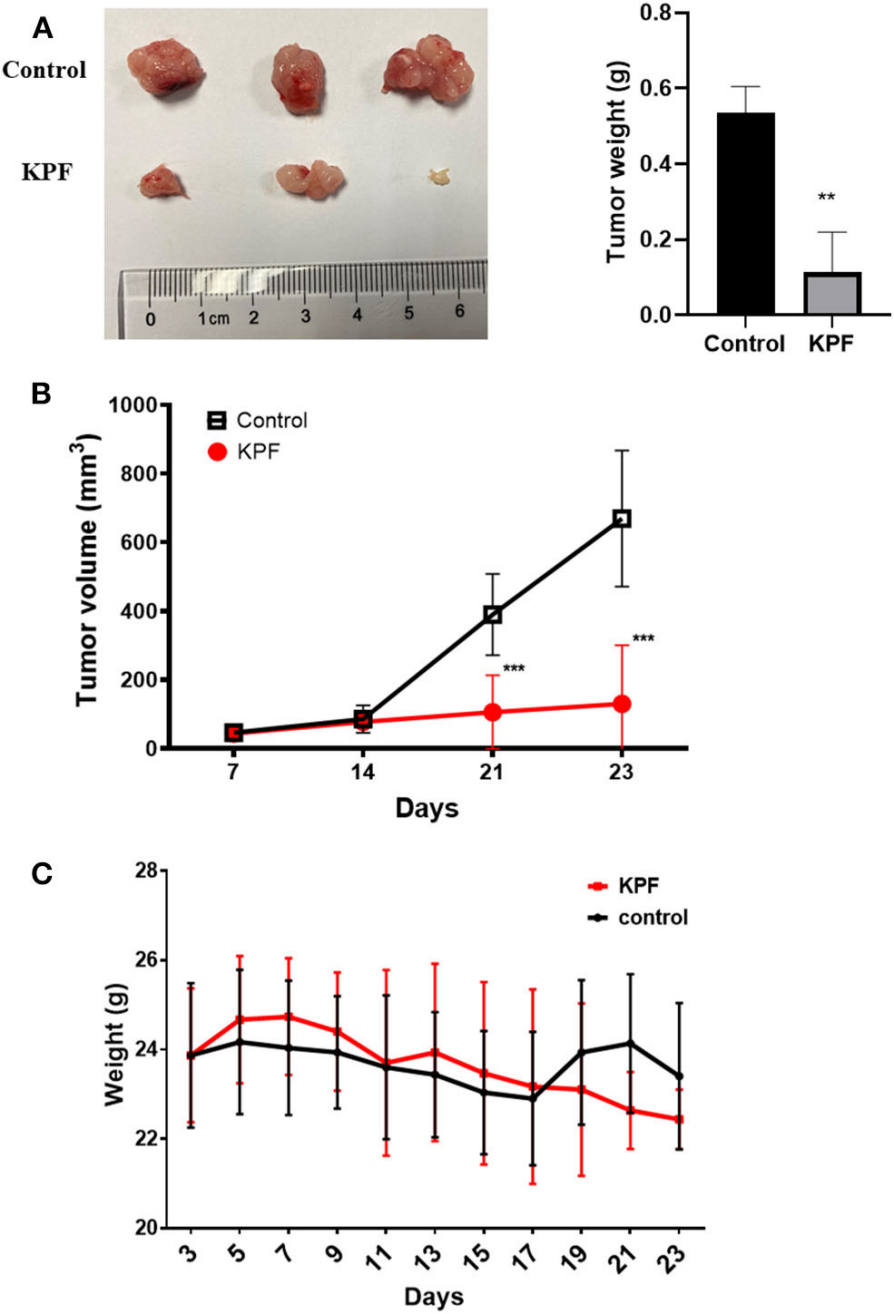
Kaempferol inhibited glioma growth *in vivo* (*n* = 3, with two biological duplication). **(A)** Tumor weight compared between control and kaempferol treatment group. **(B)** Comparison of the tumor volume between control and kaempferol treatment group. **(C)** The body weight changes of mice in control group and kaempferol treatment group were compared. Data was presented as mean ± SD. **P < 0.01 and ***P < 0.001 compared with the control.

The nude-mouse CDX model was used to investigate the anti-glioma effect of kaempferol *in vivo*. The result showed that tumor size was significantly suppressed in mice treated with kaempferol when compared with the control group (Figures 2A,B). The weight of mice was measured and found that kaempferol has no detectable impact on the weight of nude-mice at the dose of 40 mg/kg (Figure 2C).

### Kaempferol Induced Reactive Oxygen Species Generation

**Figure 3:**
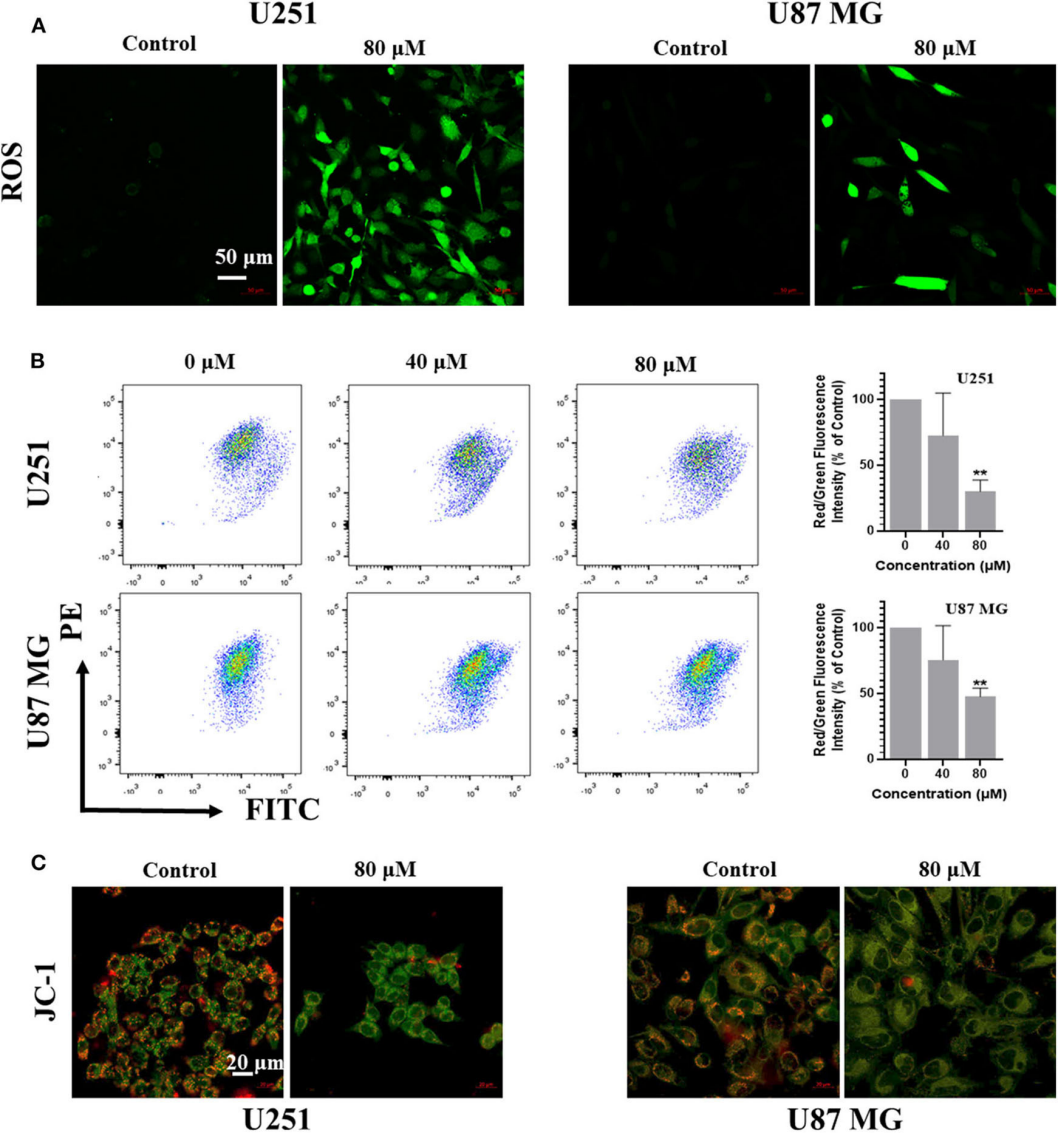
Kaempferol inhibited IL-6 secretion, induced ROS generation, and decreased mitochondrial membrane potential in glioma cells. **(A)** ROS was detected by DCFDA-H2 staining. **(B)** Mitochondrial membrane potential was measured by JC-1 probe. Red/green fluorescence intensity was measured by flow cytometry. **(C)** Fluorescence photographs of mitochondrial membrane potential in glioma cells. Data was presented as mean ± SD. **P < 0.01 compared with the control.

Reactive Oxygen Species (ROS) is one of the causes for apoptosis. Many chemotherapeutic drugs kill cancer cells by inducing ROS generation. To explore whether kaempferol increases ROS levels in GBM cells, we used fluorescent probe H2DCFDA to measure the levels of ROS in GBM cells. Our results showed that kaempferol induced ROS generation in both U87MG and U251 cells (Figure 3A). Apoptosis assay and WB result of γH2AX indicated that kaempferol could induce apoptosis via triggering DNA damage (Supplementary Figures 1A–C), which was consistent with previous reports (Sharma et al., 2007).

### Kaempferol Decreased Mitochondrial Membrane Potential in Human GBM Cells

Since ROS generation is inversely correlated with mitochondrial membrane potential by disrupting the outer mitochondrial potential to release the death-promoting proteins, we investigated mitochondrial changes by JC-1 staining. In intact mitochondria, JC-1 aggregates in the matrix of mitochondria to form polymers, which can produce red fluorescence; while the membrane potential of mitochondria is low, JC-1 is not able to gather in the matrix of mitochondria, results in the form of monomer and produce green fluorescence. A significant reduction of mitochondrial membrane potential in a dose-dependent manner was observed in kaempferol-treated U87MG and U251 cells (Figures 3B,C), indicating that kaempferol induces ROS generation and decreases mitochondrial membrane potential in GBM cells.

### Kaempferol Induced Autophagy in Human GBM Cells

**Figure 4:**
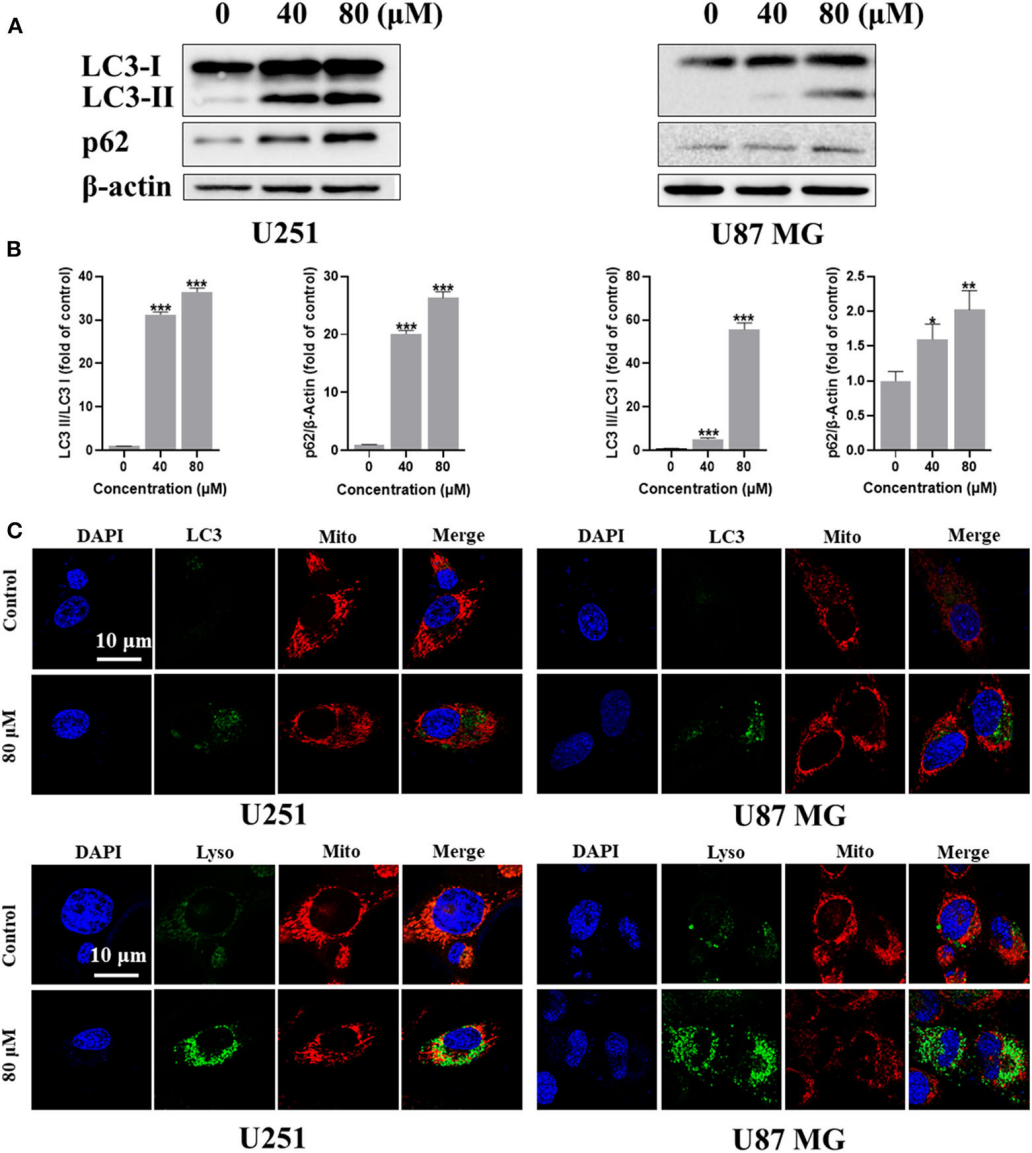
Kaempferol induced autophagy in U251 and U87 MG cells. **(A)** The protein expression of LC3-I, LC3-II, and p62 detected by Western blotting. **(B)** Immunofluorescence images of the LC3 and mitochondria. **(C)** U251 and U87 MG cells stained with MitoTracker Red and LysoTracker Green. Lyso: lysosome; and Mito: mitochondria. Data was presented as mean ± SD. *P < 0.05, **P < 0.01, ***P < 0.001 compared with the control.

The role of autophagy in tumor development is complicated, it can be tumor-suppressing or tumor-promoting at different tumor stage or tumor microenvironment condition (Amaravadi et al., 2016). Autophagy can also be triggered by ROS and cross-link with apoptosis or pyroptosis via regulating the degradation of the proteins involving in the different pathways (Su et al., 2015). To investigate whether kaempferol could induce autophagy in GBM cells, we detected the level of several autophagy markers, ubiquitin-like molecule Light chain 3 (LC3) and Sequestosome 1 (SQSTM1, also known as p62). Cleaved LC3 will further lead to form phagophore and autophagosome. The LC3-binding protein p62 is a specific substrate for autophagy. Extensive accumulation of p62 as a scaffold protein is associated with several signal pathways, including apoptosis. Immunofluorescence images showed that kaempferol treatment could increase LC3 expression in GBM cells (Figure 4A). Further quantitative analysis by western blotting showed that kaempferol treatment led to a conversion from LC3-I to LC3-II and increased the expression of p62 in a dose-dependent manner (Figure 4B), which supported that kaempferol induces autophagy in GBM cells.

Mitophagy is a form of autophagy that selectively degrades damaged or aged mitochondria, which occurred especially when the mitochondrial membrane potential decreased. Since our previous studies has confirmed that kaempferol could decrease mitochondrial membrane potential and induce autophagy, we wondered if kaempferol could induce mitophagy in GBM cells. MitoTracker Red is a red fluorescent probe, which is specifically used to stain mitochondria in living cell. LysoTracker Green is a Green fluorescent labeled lysosome probe, which can selectively stain the acid regions in living cells. LysoTracker Green and MitoTracker Red co-staining showed that kaempferol increased the expression and colocalization of lysosomes and mitochondria (Figure 4C), indicating kaempferol could induce mitophagy in GBM cells.

### Kaempferol Induced Pyroptosis in Human GBM Cells

**Figure 5:**
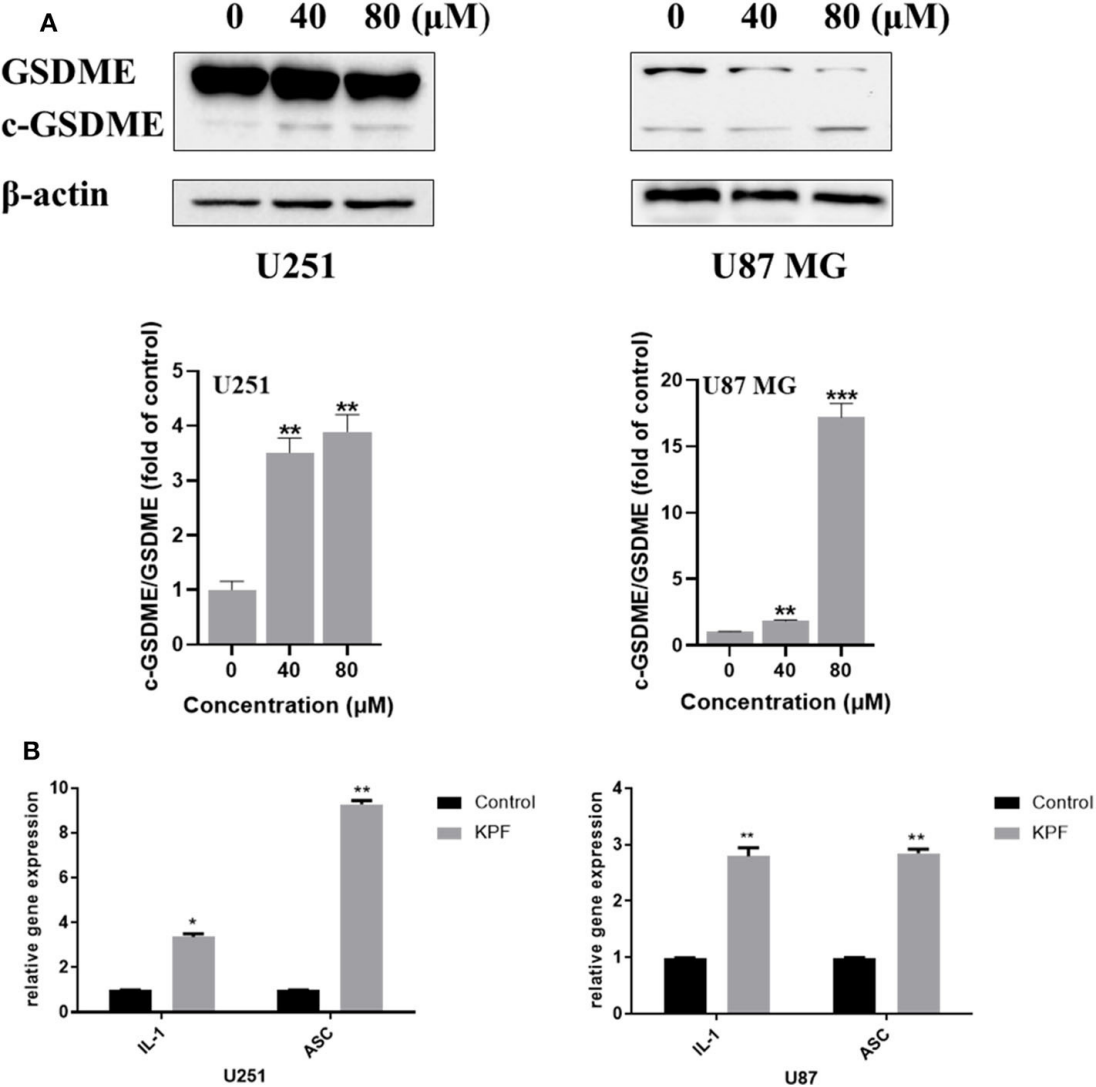
Kaempferol induced pyroptosis in glioma cells. **(A)** Western blotting showed the protein expression of GSDME, and c-GSDME in U251 and U87 MG cells. **(B)** qPCR showed the mRNA expression of IL-1β and ASC in glioma cells. Data was presented as mean ± SD. *P < 0.05, **P < 0.01, and ***P < 0.001 compared with the control.

ROS serves as an important inflammasome activation signal, while inflammasome activation could further lead to pyroptosis, a process of programmed cell death distinct from apoptosis through activation of caspase and further leading to activation of inflammatory cascade. Based on our findings that kaempferol induces ROS generation, we were curious to know whether kaempferol could induce pyroptosis in GBM cells. There are three kinds of activation pathways in pyroptosis, canonical inflammasome pathway, noncanonical inflammasome pathway and a new-found GSDME-mediated pathway. GSDME-mediated pyroptosis shares some early stage activation molecular with apoptosis, such as caspase 3. We wondered if kaempferol can induced apoptosis and trigger pyroptosis while activating the same pro-caspase3, so we detected the cleaved form of GSDME. As shown in Figure 5A, kaempferol could increase the cleavage levels of GSDME, which suggested kaempferol could induce pyroptosis in GBM cells. We also measured the mRNA expression of the proinflammatory factor IL-1β and ASC, which presented that the mRNA levels of IL-1β and ASC increased after a 24 h treatment of kaempferol (Figure 5B).

### Kaempferol Induced Pyroptosis Through Regulating Autophagy in Human GBM Cells

**Figure 6:**
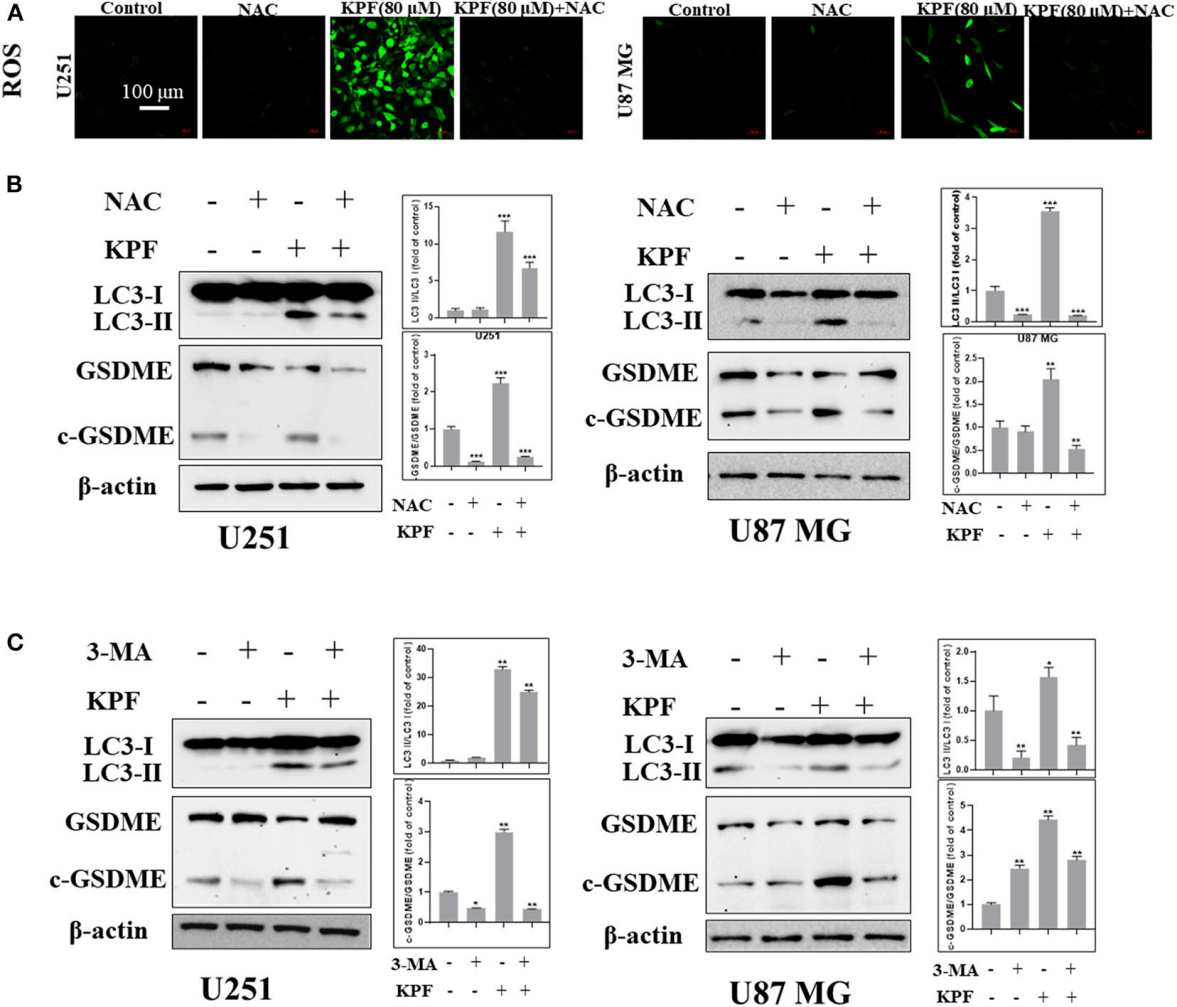
Kaempferol induced pyroptosis through regulating autophagy in glioma cells. **(A,B)** U251 and U87 MG cells were treated with or without NAC (5 mM) for 2 h before a 12 h treatment of kaempferol (80 μM). **(A)** ROS was detected by DCFDA-H2 staining. **(B)** Western blotting showed the protein expression of LC3, GSDME, and c-GSDME in glioma cells. **(C)** Glioma cells were treated with or without 3-MA (5 mM) for 2 h before a 12 h treatment of kaempferol (80 μM), the expression of LC3, GSDME, and c-GSDME was detected. Data was presented as mean ± SD. *P < 0.05, **P < 0.01, and ***P < 0.001 compared with the control.

In order to clarify the relationship between autophagy and pyroptosis, we blocked ROS generation and autophagy pathway by using antioxidant reagent N-acetyl-L-cysteine (NAC) and PI3K inhibitor 3-Methyladenine (3-MA), respectively. The result showed that NAC reduced the levels of kaempferol-inducing ROS in both U87MG and U251 cells (Figure 6A). Furthermore, we found that NAC reversed autophagy by decreasing the cleavage levels of LC3 and reversed the pyroptosis by decreasing the cleavage levels of GSDME (Figure 6B), which indicates that ROS generation induced by kaempferol contributed to autophagy and pyroptosis in GBM cells. We further used 3-MA to inhibit the pathway of autophagy, and found that the levels of cleaved form of LC3 was decreased as expected, while the levels of cleaved form of GSDME also decreased (Figure 6C), suggesting autophagy induced by kaempferol may lead to pyroptosis in GBM cells.

## Discussion

Cancer is one of the biggest challenges to human health and the second major cause of death in the world (Bray et al., 2018). Most of the current chemotherapy drugs have systemic toxicity, which limits their application in treating certain type of cancers. Increasing evidence showed that diverse compounds derived from natural products exhibited anti-cancer activities (Cragg and Pezzuto, 2016). Most of these agents are abundantly present in different plant-based food items, with low toxicity and multiple biological activities, including antioxidant, antimetastatic and anti-inflammatory activities, making them good candidates as new antitumor drugs.

Flavonoids are widely present in plants in response to microbial infection, with the structure of 2-phenylchromone and a keto carbonyl group in their molecules, flavonoids have a variety of biological activities, including antioxidant, anti-inflammatory, anti-tumor, antibacterial and antiviral activities. As one of the most common flavonoids, with broad range of activity, and low toxicity compared with other compounds, kaempferol is widely reported (Imran et al., 2019). Glioblastoma is one of the most invasive and aggressive brain tumors, most of them have high resistance against current therapies (Stupp et al., 2005). It has been reported that kaempferol inhibited both growth and migration of GBM cells, and triggered ROS generation and apoptosis (Sharma et al., 2007; Jeong et al., 2009). However, the underlying mechanisms of kaempferol in inducing glioma cell death are still elusive. Here, we verified that kaempferol could significantly inhibit glioma *in vitro* and *in vivo* via triggering ROS and further inducing autophagy and pyroptosis besides the commonly known apoptosis.

Increased generation of intracellular ROS may induce programmed cell death, such as apoptosis, autophagy and pyroptosis, via execution by lysosomal proteases or caspases (Li et al., 2015). Autophagy is a highly sensitive cellular process which can be induced in response to a wide range of stresses. A number of studies have suggested that ROS induces autophagy as upstream modulators (Filomeni et al., 2010; Szumiel, 2011). In this study, we found that inhibition of ROS can reverse the autophagy induced by kaempferol in human glioblastoma cells, indicating that ROS generation induced by kaempferol contributed to autophagy.

Pyroptosis is a type of programmed cell death with the feature of pro-inflammatory and can be triggered by increasing ROS and the subsequent activation of inflammasome-caspase-1-IL-1β signaling. GSDME belongs to the gasdermin superfamily and cleavage form of GSDME is an important marker of pyroptosis (Wang et al., 2018). In this study, we found that blocking ROS generation could reverse pyroptosis, suggesting that kaempferol induced ROS and further contributing to GSDME-mediated pyroptosis in glioma cells. Based on our current findings, kaempferol could induce ROS and further lead to both autophagy and pyroptosis. We were curious that whether pyroptosis was functionally associated with autophagy. The further results showed that inhibition of autophagy using 3-MA could reverse pyroptosis, demonstrating that pyroptosis induced by kaempferol could be regulated by autophagy in glioma cells.

In conclusion, kaempferol significantly inhibits the proliferation of GBM cells both *in vitro* and *vivo* without obvious toxicity and side effects. In addition to the common apoptosis, kaempferol cause ROS and autophagy, which further leads to pyroptosis in GBM cells. Taken together, kaempferol represents a promising new anti-glioma candidate by inducing apoptosis and triggering ROS-mediated pyroptosis through the regulation of autophagy in GBM cells.

## Data Availability Statement

The original contributions presented in the study are included in the article/Supplementary Material, further inquiries can be directed to the corresponding author/s.

## Ethics Statement

The animal study was reviewed and approved by Sun Yat-sen University.

## Author Contributions

JH and SC conceived of the original research idea. SC and JH performed the majority of experiments with the help of JM, LY, Z-QL and XC. MT helped with the animal experiments. SC and JH interpreted the data. SC and JH were responsible for the initial draft of the manuscript, whereas other authors contributed to the article and approved the submitted version.

## Conflict of Interest

The authors declare that the research was conducted in the absence of any commercial or financial relationships that could be construed as a potential conflict of interest.

## References

[B1] Amaravadi R. Kimmelman A. C. White E. (2016). Recent insights into the function of autophagy in cancer. Genes Dev 30, 1913–1930. 10.1101/gad.287524.116 27664235 PMC5066235

[B2] Bray F. Ferlay J. Soerjomataram I. Siegel R. L. Torre L. A. Jemal A. (2018). Global cancer statistics 2018: GLOBOCAN estimates of incidence and mortality worldwide for 36 cancers in 185 countries. CA Cancer J. Clin. 68, 394–424. 10.3322/caac.21492 30207593

[B3] Cragg G. M. Pezzuto J. M. (2016). Natural products as a vital source for the discovery of cancer chemotherapeutic and chemopreventive agents. Med Princ Pract. 25(Suppl 2), 41–59. 10.1159/000443404 26679767 PMC5588531

[B4] Cui Y. Morgenstern H. Greenland S. Tashkin D. P. Mao J. T. Cai L.. (2008). Dietary flavonoid intake and lung cancer–a population-based case-control study. Cancer 112, 2241–2248. 10.1002/cncr.23398 18327817 PMC5546301

[B5] Fang Y. Tian S. Pan Y. Li W. Wang Q. Tang Y.. (2020). Pyroptosis: a new frontier in cancer. Biomed. Pharmacother. 121, 109595. 10.1016/j.biopha.2019.109595 31710896

[B6] Filomeni G. Desideri E. Cardaci S. Rotilio G. Ciriolo M. R. (2010). Under the ROS…thiol network is the principal suspect for autophagy commitment. Autophagy 6, 999–1005. 10.4161/auto.6.7.12754 20639698

[B7] Galluzzi L. Pietrocola F. Bravo-San Pedro J. M. Amaravadi R. K. Baehrecke E. H. Cecconi F.. (2015). Autophagy in malignant transformation and cancer progression. EMBO J. 34, 856–880. 10.15252/embj.201490784 25712477 PMC4388596

[B8] Garcia-Closas R. Gonzalez C. A. Agudo A. Riboli E. (1999). Intake of specific carotenoids and flavonoids and the risk of gastric cancer in Spain. Cancer Causes Control 10, 71–75. 10.1023/A:1008867108960 10334645

[B9] Gilbert M. R. Wang M. Aldape K. D. Stupp R. Hegi M. E. Jaeckle K. A. (2013). Dose-dense temozolomide for newly diagnosed glioblastoma: a randomized phase III clinical trial. J. Clin. Oncol. 31, 4085–4091. 10.1200/JCO.2013.49.6968 24101040 PMC3816958

[B10] Harborne J. B. Williams C. A. (2000). Advances in flavonoid research since 1992. Phytochemistry 55, 481–504. 10.1016/S0031-9422(00)00235-1 11130659

[B11] Imran M. Rauf A. Shah Z. A. Saeed F. Imran A. Arshad M. U.. (2019). Chemo-preventive and therapeutic effect of the dietary flavonoid kaempferol: a comprehensive review. Phytother. Res. 33, 263–275. 10.1002/ptr.6227 30402931

[B12] Imran M. Salehi B. (2019). Kaempferol: a key emphasis to its anticancer *Potential* 24:2277. 10.3390/molecules24122277 31248102 PMC6631472

[B13] Jeong J. C. Kim M. S. Kim T. H. Kim Y. K. (2009). Kaempferol induces cell death through ERK and Akt-dependent down-regulation of XIAP and survivin in human glioma cells. Neurochem. Res. 34, 991–1001. 10.1007/s11064-008-9868-5 18949556

[B14] Kayagaki N. Stowe I. B. Lee B. L. O'Rourke K. Anderson K. Warming S.. (2015). Caspase-11 cleaves gasdermin D for non-canonical inflammasome signalling. Nature 526, 666–671. 10.1038/nature15541 26375259

[B15] Li L. Tan J. Miao Y. Lei P. Zhang Q. (2015). ROS and autophagy: interactions and molecular regulatory mechanisms. Cell. Mol. Neurobiol. 35, 615–621. 10.1007/s10571-015-0166-x 25722131 PMC11486209

[B16] Liu Y. Peterson D. A. Kimura H. Schubert D. (1997). Mechanism of cellular 3-(4,5-dimethylthiazol-2-yl)-2,5-diphenyltetrazolium bromide (MTT) reduction. J. Neurochem. 69, 581–593. 10.1046/j.1471-4159.1997.69020581.x 9231715

[B17] Nöthlings U. Murphy S. P. Wilkens L. R. Henderson B. E. Kolonel L. N. (2007). Flavonols and pancreatic cancer risk: the multiethnic cohort study. Am. J. Epidemiol. 166, 924–931. 10.1093/aje/kwm172 17690219

[B18] Ostrom Q. T. Gittleman H. Liao P. Vecchione-Koval T. Wolinsky Y. Kruchko C.. (2017). CBTRUS statistical report: primary brain and other central nervous system tumors diagnosed in the United States in 2010-2014. Neuro Oncol. 19, v1–v88. 10.1093/neuonc/nox158 29117289 PMC5693142

[B19] Rajendran P. Rengarajan T. Nandakumar N. Palaniswami R. Nishigaki Y. Nishigaki I. (2014). Kaempferol, a potential cytostatic and cure for inflammatory disorders. Eur. J. Med. Chem. 86, 103–112. 10.1016/j.ejmech.2014.08.011 25147152

[B20] Schroder K. Tschopp J. (2010). The inflammasomes. Cell 140, 821–832. 10.1016/j.cell.2010.01.040 20303873

[B21] Sharma V. Joseph C. Ghosh S. Agarwal A. Mishra M. K. Sen E. (2007). Kaempferol induces apoptosis in glioblastoma cells through oxidative stress. Mol. Cancer Ther. 6, 2544–2553. 10.1158/1535-7163.MCT-06-0788 17876051

[B22] Siegelin M. D. Reuss D. E. Habel A. Herold-Mende C. von Deimling A. (2008). The flavonoid kaempferol sensitizes human glioma cells to TRAIL-mediated apoptosis by proteasomal degradation of survivin. Mol. Cancer Ther. 7, 3566–3574. 10.1158/1535-7163.MCT-08-0236 19001439

[B23] Stupp R. Hegi M. E. Mason W. P. van den Bent M. J. Taphoorn M. J. Janzer R. C. (2009). Effects of radiotherapy with concomitant and adjuvant temozolomide versus radiotherapy alone on survival in glioblastoma in a randomised phase III study: 5-year analysis of the EORTC-NCIC trial. Lancet Oncol. 10, 459–466. 10.1016/S1470-2045(09)70025-7 19269895

[B24] Stupp R. Mason W. P. van den Bent M. J. Weller M. Fisher B. Taphoorn M. J. (2005). Radiotherapy plus concomitant and adjuvant temozolomide for glioblastoma. N. Engl. J. Med. 352, 987–996. 10.1056/NEJMoa043330 15758009

[B25] Su Z. Yang Z. Xu Y. Chen Y. Yu Q. (2015). Apoptosis, autophagy, necroptosis, and cancer metastasis. Mol. Cancer 14, 48 10.1186/s12943-015-0321-5 25743109 PMC4343053

[B26] Szumiel I. (2011). Autophagy, reactive oxygen species and the fate of mammalian cells. Free Radic. Res. 45, 253–265. 10.3109/10715762.2010.525233 20964552

[B27] Tsitlakidis A. Foroglou N. Venetis C. A. Patsalas I. Hatzisotiriou A. Selviaridis P. (2010). Biopsy versus resection in the management of malignant gliomas: a systematic review and meta-analysis. J. Neurosurg. 112, 1020–1032. 10.3171/2009.7.JNS09758 19747048

[B28] Wang Y. Gao W. Shi X. Ding J. Liu W. He H.. (2017). Chemotherapy drugs induce pyroptosis through caspase-3 cleavage of a gasdermin. Nature 547, 99–103. 10.1038/nature22393 28459430

[B29] Wang Y. Yin B. Li D. Wang G. Han X. Sun X. (2018). GSDME mediates caspase-3-dependent pyroptosis in gastric cancer. Biochem. Biophys. Res. Commun. 495, 1418–1425. 10.1016/j.bbrc.2017.11.156 29183726

[B30] Wen P. Y. Kesari S. (2008). Malignant gliomas in adults. N. Engl. J. Med. 359, 492–507. 10.1056/NEJMra0708126 18669428

