# Supplementary material for: Anti-glioblastoma Activity of Kaempferol via Programmed Cell Death Induction: Involvement of Autophagy and Pyroptosis
Source: Front Bioeng Biotechnol. 2020 Dec 10;8:614419. doi: 10.3389/fbioe.2020.614419 (PMC7758214; doi:10.3389/fbioe.2020.614419)
Supplement: Supplementary file 1 [file Data_Sheet_1.PDF]

Supplementary Material

1 Supplementary Figures

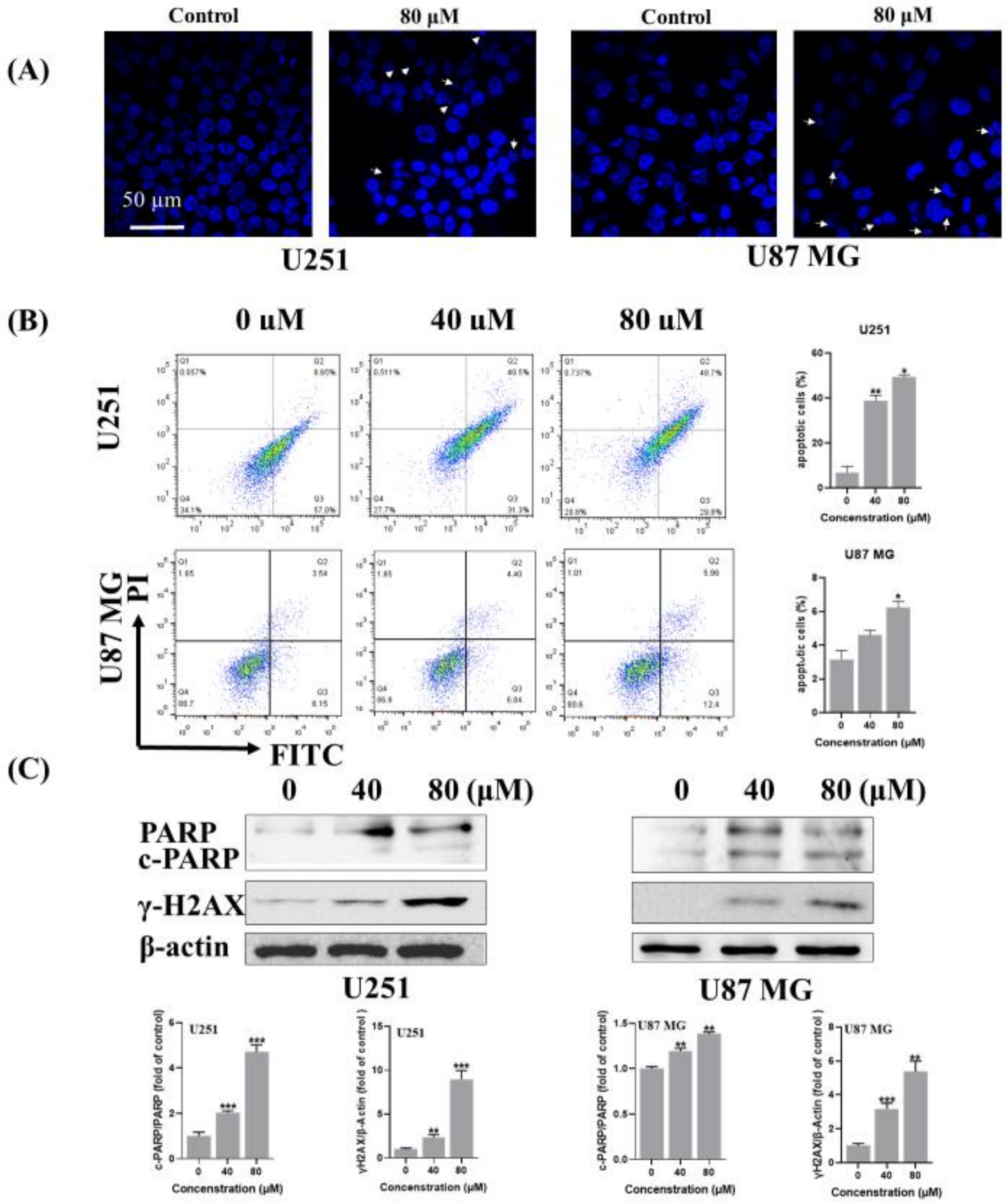

Supplementary Figure 1. Kaempferol induced apoptosis by increasing DNA damage in glioma cells. **(A)** Hoechst staining representated the apoptotic cells. **(B)** Flow cytometry showed the apoptotic cells. **(C)** The expression of cleaved PARP and γH2AX in U251 and U87 MG cells was analyzed by Western blotting. Data was presented as mean ± SD. *$P < 0.05$, **$P < 0.01$, ***$P < 0.001$ compared with the control.
